# Phosphatidylcholine coordinates ER-autonomous and ER-nonautonomous adaptations to unfolded protein response dysfunction

**DOI:** 10.1016/j.jbc.2025.111026

**Published:** 2025-12-07

**Authors:** Haixiang Tong, Wei Li, Pangui Yuan, Xinyu Wang, Shanshan Pang, Haiqing Tang

**Affiliations:** School of Life Sciences, Chongqing University, Chongqing, China

**Keywords:** UPR, lysosome, phosphatidylcholine, proteostasis, aging

## Abstract

The ER UPR plays a crucial role in maintaining proteostasis, with its dysfunction closely associated with aging and various diseases. However, how cells cope with ER UPR dysfunction remains largely unexplored. Here, we report that both ER-autonomous and ER-nonautonomous adaptive responses are activated by defects in the IRE-1/XBP-1 UPR branch in *Caenorhabditis elegans*. IRE-1/XBP-1 dysfunction not only triggers the activation of the PEK-1 UPR branch but also induces a lysosome-dependent cytosolic proteostatic response. Mechanistically, IRE-1/XBP-1 dysfunction downregulates phosphatidylcholine (PC) metabolism, reducing levels of membrane lipid PC. This PC deficiency drives BORC complex recruitment to lysosomes, triggering lysosomal activation. Furthermore, suppression of phosphatidylcholine metabolism alone sufficiently activates both the ER UPR and lysosomal pathways, thereby enhancing resilience to proteostatic stress and contributing to longevity. These findings provide insights into how cells integrate distinct adaptive responses to maintain systemic proteostasis when the ER UPR is compromised and identify phosphatidylcholine as a potent regulator of proteostasis and aging.

Protein quality control is crucial for cellular function and healthy aging. To maintain proteostasis, organisms have evolved sophisticated surveillance mechanisms, including the Unfolded Protein Response (UPR) in the endoplasmic reticulum (ER), which monitors protein folding stress through three key pathways: IRE1, PERK, and ATF6 (1). Defective UPR function is closely linked with aging and various human diseases (2, 3, 4). For example, aging is associated with a notable decline in the function of the IRE1 and PERK pathways, leading to UPR failure and proteostatic imbalance (5). Notably, activation of the UPR during aging in the model organism *Caenorhabditis elegans* has been shown to improve proteostasis and extend lifespan (6, 7), highlighting the crucial role of the ER UPR in maintaining healthy aging. Given the essential role of UPR in cellular homeostasis and animal health, we hypothesize that cells have evolved adaptive mechanisms to maintain proteostasis when UPR function is compromised.

The three UPR pathways exhibit some functional redundancy, allowing dysfunction in one pathway to trigger compensatory activation of another. For example, inhibition of the IRE1 pathway in *C. elegans* activates the PERK pathway (8), representing an ER-autonomous adaptive response, although the regulatory mechanisms underlying this adaptation remain unclear.

Proteostatic stress in the ER also impacts other cellular compartments (9, 10, 11), as many proteins destined for these compartments depend on ER folding and transport. Consequently, UPR activation during ER stress not only restores ER proteostasis but also stimulates cytoplasmic protein quality control machinery, such as autophagy (12). This dual role suggests that ER UPR dysfunction poses a significant threat to overall cellular homeostasis. A key question then emerges: in the context of ER UPR decline, can cytoplasmic protein quality control systems be adaptively activated to maintain global proteostasis? If so, is this ER-nonautonomous adaptation related to ER-autonomous response?

In this study, we explored cellular adaptations to UPR dysfunction using *C. elegans* as a model organism. Our findings reveal that dysfunction of the IRE-1/XBP-1 pathway activates lysosomal degradation as an ER-nonautonomous adaptive response. Remarkably, both ER-autonomous and ER-nonautonomous adaptive responses are orchestrated by the membrane lipid phosphatidylcholine (PC). This coordination highlights how cells integrate diverse adaptive mechanisms to maintain systemic proteostasis. Furthermore, we demonstrate that reducing PC levels is sufficient to enhance proteostasis, increase resilience to proteostatic stress, and extend lifespan.

## Results

### *Xbp-1* deficiency improves cytosolic proteostasis

**Figure 1:**
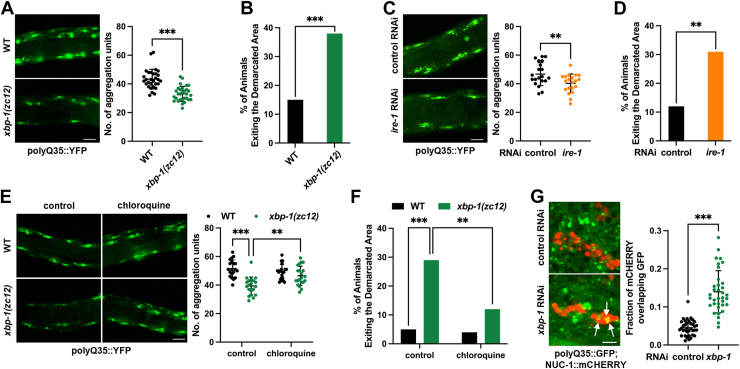
***ire-1/xbp-1* deficiency improves cytosolic proteostasis.***A*, effect of *xbp-1(zc12)* mutation on cytosolic polyQ35::YFP aggregation in day 8 adults. n = 30 animals. *B*, effect of *xbp-1(zc12)* mutation on motility in polyQ35::YFP day 8 adults. n = 80 animals. *C*, effect of *ire-1* RNAi on cytosolic polyQ35::YFP aggregation in day 8 adults. n = 20 animals. *D*, effect of *ire-1* RNAi on motility in polyQ35::YFP day 8 adults. n = 80 animals. *E*, effect of chloroquine supplementation on cytosolic polyQ35::YFP aggregation in day 8 *xbp-1(zc12)* mutants. n = 20 animals. *F*, effect of chloroquine supplementation on motility in polyQ35::YFP day 8 adults with the *xbp-1(zc12)* mutation. n = 80 animals. *G*, effect of *xbp-1* RNAi on colocalization between polyQ35::GFP and NUC-1::mCHERRY in the hypodermis. n = 30 animals. White arrows indicate colocalization sites. Data are presented as mean ± SD. ∗∗*p* < 0.01, ∗∗∗*p* < 0.001. Scale bar = 50 μm for panels (*A*, *C*, and *E*), Scale bar = 2 μm for panel (*G*). *A*, *C*, and *G*, were analyzed by unpaired two-tailed *t* test. *B*, *D*, and *F*, were analyzed by Chi-square and Fisher’s exact test. *E*, was analyzed by two-way ANOVA with Tukey’s multiple comparisons test.

We first explored how defects in the ER UPR might affect cytosolic proteostasis. To assess this, we examined the levels of cytosolic protein aggregation in response to mutations in key UPR genes in *C. elegans*, specifically *xbp-1/Xbp1*, *perk-1/Perk*, and *atf-6/Atf6*. Polyglutamine (polyQ) protein aggregation is a well-established reporter of *C. elegans* cytosolic proteostasis (13). For instance, PolyQ35::YFP is typically diffusely distributed in the cytosol of young adult worms but forms foci as animals age, reflecting increased protein aggregation and compromised cytosolic proteostasis (14). While ER UPR activation is believed to improve cytosolic proteostasis (12, 15, 16), we found that none of the UPR mutants exacerbates polyQ35::YFP aggregation in aged animals (Figs. 1*A*, and S1, *A* and *B*). Surprisingly, the *xbp-1* mutation even greatly improved it, as *xbp-1* mutants displayed a marked reduction in YFP foci compared to wild-type (WT) controls in aged animals (Fig. 1*A*). The *xbp-1* mutation used was *zc12*, which is a C-to-T change that generates a premature stop codon and is considered a null allele (17). Since the polyQ aggregation in muscle cells is known to impair motility (14), we also assessed locomotor activity. Aged *xbp-1* mutants demonstrated improved motility (Fig. 1*B*), an effect not observed in *pek-1* or *atf-6* mutants (Fig. S1, *C* and *D*). Similar improvements in cytosolic proteostasis and motility were observed when *ire-1* was silenced by RNAi (Fig. 1, *C* and *D*). Together, these data suggest that defects in the IRE-1/XBP-1 pathway enhance cytosolic proteostasis in *C. elegans*.

### *Xbp-1* deficiency improves cytosolic proteostasis *via* lysosomes

Cytosolic proteostasis relies on several protein quality control mechanisms. Lysosomes play a critical role in maintaining cytosolic proteostasis by degrading protein aggregates (18), complementing proteasome-mediated degradation (19). Additionally, proper protein folding, regulated by cytosolic heat shock proteins (HSPs), is essential for proteostasis (20). We hypothesized that one or more of these pathways contribute to the improved cytosolic protein aggregation observed in *xbp-1* mutants. To test this, we independently silenced each pathway and assessed polyQ35::YFP aggregation in *xbp-1* mutants. First, we treated *xbp-1* mutants with the proteasome inhibitor bortezomib and observed that *xbp-1* mutants retained enhanced cytosolic proteostasis (Fig. S1, *E* and *F*), suggesting that the proteasome does not contribute to the proteostatic regulation in *xbp-1* mutants. Next, we silenced *hsf-1*, the master regulator of cytosolic HSPs (20). Even with *hsf-1* RNAi, *xbp-1* deficiency still significantly reduced polyQ35::YFP foci and improved motility (Fig. S1, *G* and *H*). Moreover, *xbp-1* deficiency did not induce the expression of cytosolic HSPs (Fig. S1*I*). These data suggest that cytosolic HSPs are not required for improved cytosolic protein aggregation in *xbp-1*-deficient animals.

We then treated *xbp-1* mutants with chloroquine, a lysosomal inhibitor, and found that it abolished the enhanced cytosolic proteostasis observed in *xbp-1* mutants (Fig. 1, *E* and *F*), suggesting the essential role of lysosomal function in the proteostatic adaptation driven by *xbp-1* deficiency. We therefore hypothesized that lysosomes mediate polyQ35 aggregate degradation, which should increase their association with these aggregates. Indeed, by using a well-established *C. elegans* lysosomal marker NUC-1::mCHERRY (21), we observed that while polyQ35 foci showed minimal association with lysosomes in WT animals, *xbp-1* deficiency significantly increased the colocalization of polyQ35 aggregates with lysosomes (Fig. 1*G*). Collectively, our data imply that *xbp-1* deficiency may trigger a lysosome-dependent mechanism to sustain cytosolic proteostasis.

### *Xbp-1* deficiency activates lysosomal degradation

**Figure 2:**
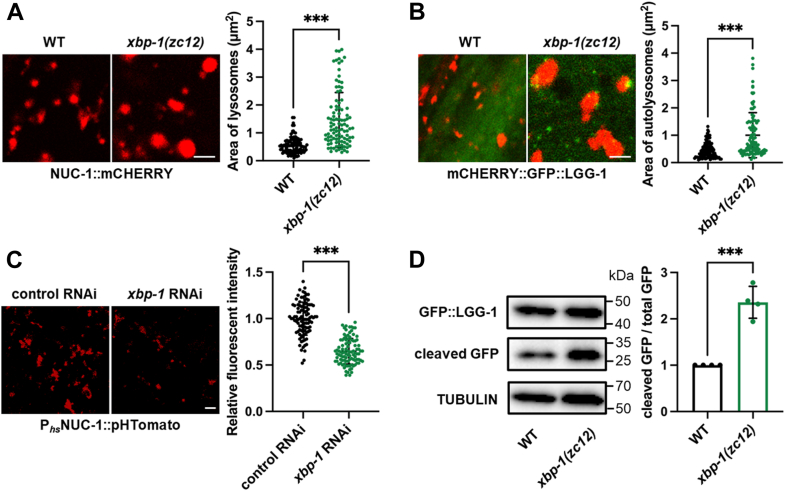
***xbp-1* deficiency activates lysosomes.***A*, effect of *xbp-1(zc12)* mutation on lysosomal morphology and size in day 1 adults. n = 100 lysosomes from 6 worms per group. *B*, effect of *xbp-1(zc12)* mutation on the size of ALs in day 1 adults, as indicated by red fluorescence of mCHERRY::GFP::LGG-1. n = 100 ALs from 6 worms per group. *C*, effect of *xbp-1* RNAi on lysosomal acidity in day 1 adults. n = 100 lysosomes from 6 worms per group. *D*, effect of *xbp-1(zc12)* mutation on GFP::LGG-1 cleavage in day 1 adults. n = 4 independent experiments. Data are presented as mean ± SD. ∗∗∗*p* < 0.001. Scale bar = 2 μm. *A*–*D*, were analyzed by unpaired two-tailed *t* test.

We next investigated lysosomal phenotypes in *xbp-1* mutants, beginning with lysosomal morphology, which is closely linked to their function (22, 23). NUC-1 is a lysosomal DNase, and the NUC-1::mCHERRY fusion protein serves as a well-established lysosomal reporter (21). Using this reporter, we observed a marked increase in lysosomal size in *xbp-1* mutants and RNAi-treated animals (Figs. 2*A* and S2*A*). Lysosomes play an essential role in clearing cytosolic protein aggregates through autophagy by fusing with autophagosomes to form autolysosomes (ALs), where the aggregates are degraded (18). EPG-5 mediates autophagosome-lysosome fusion in *C. elegans* (24). Notably, *epg-5* RNAi reduced lysosomal enlargement in *xbp-1* mutants, suggesting that the degradative ALs are also enlarged in these animals (Fig. S2*B*). To further corroborate this, we employed the mCHERRY::GFP::LGG-1 reporter. LGG-1 is an autophagosome marker protein, and in this reporter, mCHERRY selectively labels ALs after autophagosome-lysosome fusion, as GFP fluorescence is quenched in acidic ALs (25). This reporter confirmed the presence of enlarged ALs in *xbp-1* mutants (Fig. 2*B*). These data collectively suggest that *xbp-1* mutation alters lysosomal morphology.

We next examined lysosomal function. To measure lysosomal acidity, we utilized the NUC-1::pHTomato fluorescent protein, which exhibits increased fluorescence at elevated pH levels (26). In *xbp-1* deficient animals, we observed a reduction in NUC-1::pHTomato fluorescence, indicating enhanced lysosomal acidity (Fig. 2*C*). As a control, *xbp-1* deficiency did not alter the fluorescence intensity of the pH-insensitive NUC-1::mCHERRY (Fig. S2*C*), suggesting that the observed decrease in pHTomato signal reflects increased acidity rather than reduced NUC-1 expression. While lysosomal acidity is crucial for enzymatic activity, the *xbp-1* mutation did not appear to improve lysosomal enzyme activity, as measured by the cleavage of NUC-1::mCHERRY (Fig. S2*D*). In this assay, mCHERRY cleavage by lysosomal cathepsins serves as an indicator of enzyme activity. We then examined whether the enlarged lysosomes in *xbp-1* mutants exhibit enhanced degradation capacity. To test this, we measured the cleavage of GFP::LGG-1 (27), where GFP cleavage within ALs serves as an indicator of AL degradation capacity. The results demonstrated that AL degradation capacity was enhanced in *xbp-1* mutants (Fig. 2*D*). Additionally, we employed the Magic Red staining assay, which indicates the lysosomal degradation capacity by assessing red fluorescence released through lysosomal cathepsin cleavage. This assay further confirmed the enhanced lysosomal degradation in *xbp-1* mutants (Fig. S2*E*). Together, these findings suggest that *xbp-1* mutation leads to enlarged lysosomes with enhanced degradation capacity.

### The membrane lipid PC mediates lysosomal activation in *xbp-1* mutants

**Figure 3:**
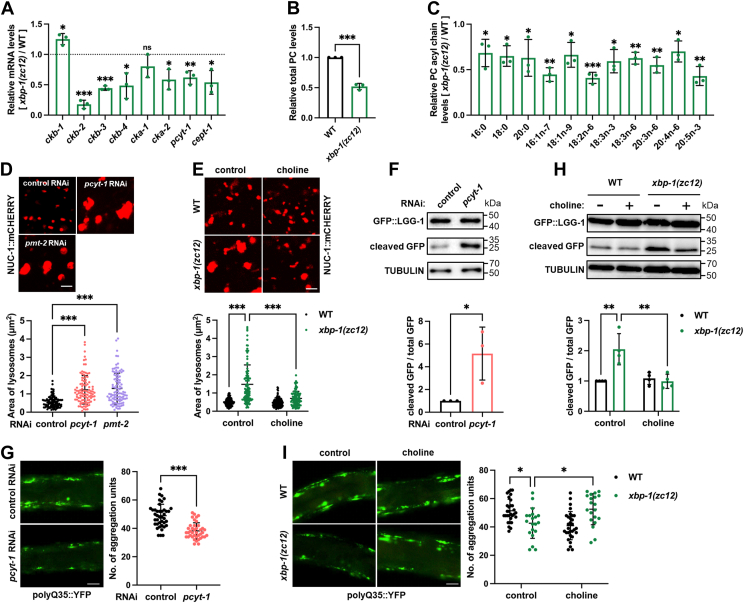
**PC mediates lysosomal activation and cytosolic proteostasis in *xbp-1* mutants.***A*, effect of *xbp-1(zc12)* mutation on the mRNA expression of PC metabolic enzymes in day 1 adults. n = 3 independent experiments. *B* and *C*, effect of *xbp-1(zc12)* mutation on the levels of total PC (*B*) and PC acyl chains (*C*) in day 1 adults. n = 3 independent experiments. *D*, effects of *pcyt-1* and *pmt-2* RNAi on lysosomal morphology and size in day 1 adults. n = 100 lysosomes from 6 worms per group. *E*, effect of choline supplementation on lysosomal morphology and size in day 1 *xbp-1(zc12)* mutants. n = 100 lysosomes from 6 worms per group. *F*, effects of *pcyt-1* RNAi on GFP::LGG-1 cleavage in day 1 adults. n = 3 independent experiments. *G*, effect of *pcyt-1* RNAi on cytosolic polyQ35::YFP aggregation in day 8 adults. n = 40 animals. *H*, effect of choline supplementation on GFP::LGG-1 cleavage in day 1 *xbp-1(zc12)* mutants. n = 4 independent experiments. *I*, effect of choline supplementation on cytosolic polyQ35::YFP aggregation in day 8 *xbp-1(zc12)* mutants. n = 20 to 30 animals. Data are presented as mean ± SD. ∗*p* < 0.05, ∗∗*p* < 0.01, ∗∗∗*p* < 0.001. Scale bar = 2 μm for panels (*D* and *E*); 50 μm for panels (*G* and *I*). *A* and *C*, were analyzed by Multiple *t* test with correction for multiple comparisons using the Holm–Sidak method. *B*, *F*, and *G*, were analyzed by unpaired two-tailed *t* test. *D*, was analyzed by one-way ANOVA with Dunnett’s multiple comparisons test. *E*, *H*, and *I*, were analyzed by two-way ANOVA with Tukey’s multiple comparisons test.

Lipids are emerging as crucial regulators of inter-organelle communication (28). The ER plays a central role in maintaining lipid homeostasis alongside its well-established function in protein homeostasis (29). The ER UPR can be activated under conditions of membrane lipid disequilibrium, commonly referred to as lipid bilayer stress (30, 31, 32, 33). In *C. elegans*, this stress is induced by a reduction in membrane lipid PC, which activates the IRE-1/XBP-1 branch of the ER UPR (15, 34). Thus, cells with reduced IRE-1/XBP-1 activity might interpret this as a state of PC abundance and suppress PC metabolism accordingly. Consistent with this hypothesis, *xbp-1* mutants exhibited significantly downregulated expression of several PC metabolic enzymes (Fig. 3*A*), accompanied by reduced PC levels (Fig. 3, *B* and *C*). Notably, while the PEK-1 branch coregulates numerous downstream genes redundantly with IRE-1/XBP-1 branch (35), *pek-1* mutants showed no alteration in PC metabolic gene expression (Fig. S3*A*), demonstrating specific regulation of PC metabolism by IRE-1/XBP-1 branch. We also measured other lipids in *xbp-1* mutants, including phosphatidylethanolamine (PE) and triglyceride (TAG), and found their levels to be unchanged (Fig. S3, *B* and *D*). Given that lysosomal homeostasis is tightly regulated by various lipid species (36, 37), we speculated that the reduction of PC in *xbp-1* mutants might contribute to lysosomal activation.

In *C. elegans*, PC is synthesized either from choline *via* Kennedy pathway by enzymes like phosphocholine cytidylyltransferase-1 (PCYT-1) or through a series of methylations by PMT-1/PMT-2 from phosphoethanolamine (38, 39) (Fig. S3*E*). RNAi-mediated knockdown of *pcyt-1* (Fig. S3, *F* and *G*) or *pmt-2* (40) efficiently reduced PC levels. The acyl chains regulated by *xbp-1* mutation (Fig. 3*C*) closely resembled those affected by *pcyt-1* RNAi (Fig. S3*G*), but not by *pmt-2* RNAi (40), suggesting that XBP-1 regulates PC content through the PCYT-1-dependent Kennedy pathway.

Strikingly, RNAi targeting *pcyt-1* or *pmt-2*, akin to *xbp-1* mutation, resulted in the enlargement of lysosomes (Fig. 3*D*) and degradative ALs (Fig. S4*A*). The *sams-1* gene, which acts upstream of *pmt-2* in PC synthesis (Fig. S3*E*), also played a similar role, as *sams-1* mutants displayed enlarged lysosomes (Fig. S4*B*). Critically, when we restored PC levels in *xbp-1* mutants through supplementation of choline, the precursor for PC synthesis (Fig. S3*E*), we not only confirmed PC recovery (Fig. S4*C*) but also observed complete reversal of lysosomal enlargement (Fig. 3*E*), demonstrating that PC reduction regulates lysosomal morphology in response to *xbp-1* deficiency.

We next assessed the role of PC in lysosomal function and cytosolic protein aggregation in *xbp-1* mutants. In WT animals, *pcyt-1* RNAi enhanced lysosomal degradation of GFP::LGG-1 (Fig. 3*F*), increased the colocalization of polyQ35::YFP foci with lysosomes (Fig. S4*D*), reduced polyQ35::YFP aggregation (Fig. 3*G*), and improved animal motility (Fig. S4*E*). These findings suggest that PC reduction is sufficient to improve lysosomal function and cytosolic proteostasis. Conversely, in *xbp-1*-deficient animals, choline supplementation abolished the enhanced GFP::LGG-1 cleavage (Fig. 3*H*), attenuated polyQ35::YFP-lysosome colocalization (Fig. S4*F*), blunted the reduction in polyQ35::YFP aggregation (Fig. 3*I*), and reversed the associated motility improvement (Fig. S4*G*). Additionally, we also tested the effects of choline supplementation on *xbp-1;pcyt-1(RNAi)* double deficient animals. We anticipated that choline would have no rescuing effects in this background, as the generation of PC from choline requires PCYT-1 (Fig. S3*E*). As expected, choline supplementation did not reverse the enlarged lysosomes, enhanced autophagic flux, and improved cytosolic proteostasis in *xbp-1;pcyt-1(RNAi)* animals (Fig. S4, *H*–*J*). These findings collectively suggest that *xbp-1* mutation activates lysosomes and improves cytosolic proteostasis *via* PC modulation.

### BORC mediates lysosomal activation downstream of PC

We next investigated the mechanism by which reduced PC levels activate lysosomes. A feature of lysosomes in *C. elegans* with *xbp-1* mutation or PC deficiency is their enlarged size (Fig. 3). The BLOC-1-related complex (BORC) is known to regulate lysosomal size in mammalian cells. BORC associates with the lysosomal membrane *via* its myrlysin subunit, thereby restricting lysosomal fission and promoting enlargement (41, 42). We therefore tested whether BORC acts as a downstream effector of PC to regulate lysosomes.

**Figure 4:**
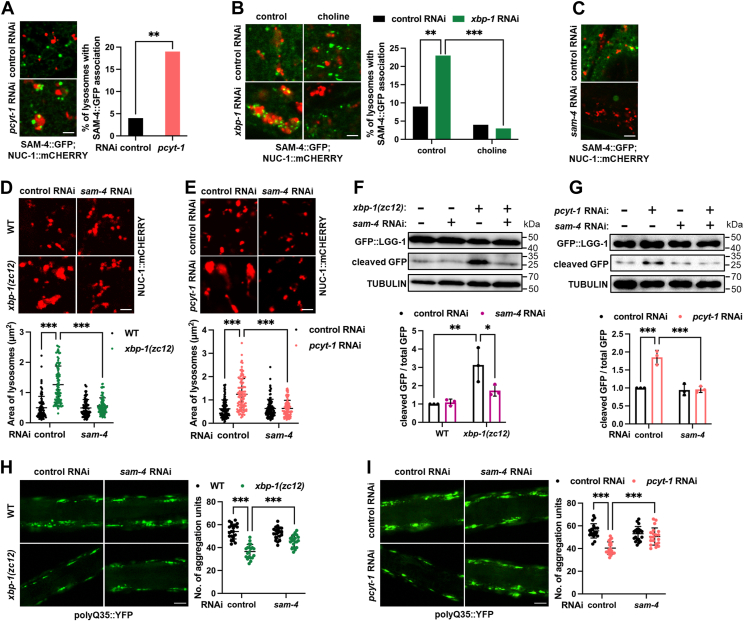
**SAM-4 mediates lysosomal activation downstream of PC.***A*, effect of *pcyt-1* RNAi on the association between lysosomes (NUC-1::mCHERRY) and SAM-4::GFP in day 1 adults. n = 100 lysosomes from 6 worms per group. *B*, effect of choline supplementation on the association between lysosomes and SAM-4::GFP under *xbp-1* deficiency in day 1 adults. n = 100 lysosomes from 6 worms per group. *C*, effect of *sam-4* RNAi on SAM-4::GFP. *D* and *E*, effect of *sam-4* RNAi on lysosomal morphology and size under *xbp-1* deficiency (*D*) and *pcyt-1* deficiency (*E*) in day 1 adults. n = 100 lysosomes from 6 worms per group. *F* and *G*, Effect of *sam-4* RNAi on GFP::LGG-1 cleavage under *xbp-1* deficiency (*F*) and *pcyt-1* deficiency (*G*) in day 1 adults. n = 3 independent experiments. *H* and *I*, effect of *sam-4* RNAi on cytosolic polyQ35::YFP aggregation in day 8 adults under *xbp-1* deficiency (*H*) and *pcyt-1* deficiency (*I*). n = 20 animals. Data are presented as mean ± SD. ∗*p* < 0.05, ∗∗*p* < 0.01, ∗∗∗*p* < 0.001. Scale bar = 2 μm for panels (*A*–*E*); 50 μm for panels (*H* and *I*). *A* and *B*, were analyzed by Chi-square and Fisher’s exact test. *D*–*I*, were analyzed by two-way ANOVA with Tukey’s multiple comparisons test.

To address this question, we first examined whether a reduction in membrane lipid PC levels alters BORC-lysosome association. SAM-4 is the *C. elegans* ortholog of myrlysin. By constructing a SAM-4::GFP reporter, we found that *pcyt-1* RNAi significantly enhanced the association between lysosomes and SAM-4 (Fig. 4*A*). Moreover, *xbp-1* RNAi also increased their association, which was reversed by choline supplementation (Fig. 4*B*). These results indicate that reduced PC levels in *xbp-1*-deficieny animals promote BORC association with lysosomes.

We then tested the requirement of SAM-4 for PC-dependent lysosomal phenotypes in *xbp-1* mutants. *sam-4* RNAi efficiently depleted SAM-4::GFP signal, confirming RNAi efficacy (Fig. 4*C*). Consistent with BORC’s role as a lysosomal size regulator, *sam-4* RNAi abolished lysosomal enlargement in both *xbp-1* mutants (Fig. 4*D*) and PC-deficient animals (Fig. 4*E*). Moreover, *sam-4* RNAi suppressed the enhanced lysosomal degradation capacity (Fig. 4, *F* and *G*) and reversed the amelioration of cytosolic protein aggregation observed in these animals (Fig. 4, *H* and *I*). Collectively, these results establish that following *xbp-1* mutation, a decrease in the membrane lipid PC promotes the recruitment of the BORC complex to lysosomes. This, in turn, alters lysosomal morphology and enhances lysosomal activity.

### Aging-associated lysosomal changes is regulated by PC

**Figure 5:**
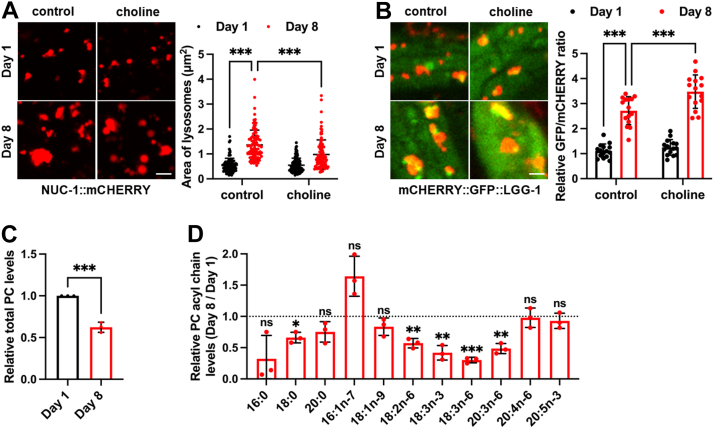
**PC reduction modulates aging-associated lysosomal changes.***A*, effect of choline supplementation on vesicular lysosomal size during aging. n = 100 lysosomes from 6 worms per group. *B*, effect of choline supplementation on the GFP/mCHERRY ratio of mCHERRY::GFP::LGG-1 worms during aging. n = 15 animals. *C*, the levels of total PC and PC acyl chains in day 1 and day 8 WT animals. n = 3 independent experiments. Data are presented as mean ± SD. ∗*p* < 0.05, ∗∗*p* < 0.01, ∗∗∗*p* < 0.001. Scale bar = 2 μm for *panels* (*A*); 1 μm for *panels* (*B*). *A* and *B*, were analyzed by two-way ANOVA with Tukey’s multiple comparisons test. *C*, was analyzed by unpaired two-tailed *t* test. *D*, was analyzed by Multiple *t* test with correction for multiple comparisons using the Holm–Sidak method.

Aging in *C. elegans* is associated with a functional decline in the IRE-1/XBP-1 branch (6). We therefore asked whether PC and lysosomal changes observed in *xbp-1* mutants might also occur in normal aging. While aging has previously been linked to an increase in tubular lysosomes (26), we found that it was also associated with an enlargement of vesicular lysosomes (Fig. 5*A*), resembling the phenotype seen in *xbp-1* mutants. We then assessed vesicular lysosomal activity during aging by employing *C. elegans* expressing the mCHERRY::GFP::LGG-1 autophagy reporter. LGG-1 is an AP protein and GFP fluorescence is quenched in acidic environment after AP fusion with lysosomes, thus a decrease of the GFP/mCHERRY ratio indicates enhanced autophagic flux efficiency. Using this assay, we observed an age-related decline in lysosomal function (Fig. 5*B*).

We then measured PC content and found that PC levels decrease with age (Fig. 5, *C* and *D*). We hypothesized that if PC reduction in aging, as in *xbp-1* mutants, exerts a protective effect on lysosomal function, then choline supplementation should worsen lysosomal function in aged animals. As predicted, choline not only abolished the age-associated enlargement of vesicular lysosomes (Fig. 5*A*) but also further impaired autophagic flux (Fig. 5*B*). These data suggest that, similar to the case in *xbp-1* mutants, PC reduction during aging may also act as an adaptive response that helps maintain lysosomal function.

### ER-autonomous response to UPR deficiency is mediated by PC

**Figure 6:**
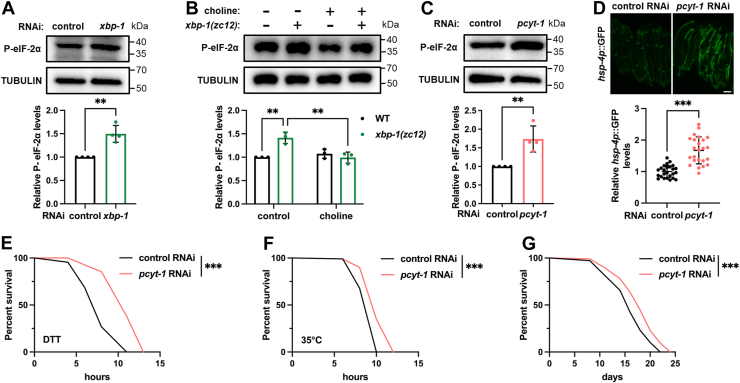
**PC reduction is sufficient to enhance stress resilience and extend lifespan.***A*, effect of *xbp-1* RNAi on the phosphorylation of eIF-2α in day 1 adults. n = 4 independent experiments. *B*, effect of choline supplementation on the phosphorylation of eIF-2α in day 1 *xbp-1(zc12)* mutants. n = 3 independent experiments. *C*, effect of *pcyt-1* RNAi on the phosphorylation of eIF-2α in day 1 adults. n = 4 independent experiments. *D,* effect of *pcyt-1* RNAi on the expression of *hsp-4p*::GFP in day 1 adults. n = 25 animals. *E* and *F*, effect of *pcyt-1* RNAi on dithiothreitol (DTT) resistance (*E*) and heat shock resistance (*F*). *G*, effect of *pcyt-1* RNAi on lifespan. Data are presented as mean ± SD. ∗∗*p* < 0.01, ∗∗∗*p* < 0.001. Scale bar = 200 μm for *panel* (*D*). *A*, *C*, and *D*, were analyzed by unpaired two-tailed *t* test. *B*, was analyzed by two-way ANOVA with Tukey’s multiple comparisons test. *E*–*G*, were analyzed by log-rank (Mantel–Cox) test. Additional repeats and statistical analyses for survival data were provided in Table S1.

The PEK-1 branch of the ER UPR co-regulates numerous downstream genes in coordination with the IRE-1/XBP-1 branch, with functional redundancy (35, 43). Moreover, it has been shown that IRE-1/XBP-1 deficiency can activate PEK-1 as a compensatory mechanism (8). We confirmed that *xbp-1* knockdown resulted in elevated eIF-2α phosphorylation, indicating PEK-1 activation (Fig. 6*A*). This finding suggests that, in addition to lysosomal activation, *xbp-1* deficiency also triggers an ER-autonomous adaptive response. We further asked whether PC also mediates this ER-autonomous response and found that choline supplementation completely abrogated PEK-1 activation induced by *xbp-1* mutation (Fig. 6*B*). This result suggests that both ER-autonomous and ER-nonautonomous adaptive responses are coordinated by PC modulation.

### PC reduction is sufficient to enhance animal stress resilience and extend lifespan

Given the critical role of PC in both ER-autonomous and ER-nonautonomous adaptive responses, we hypothesized that the reduction of PC alone could simultaneously trigger both ER and cytosolic proteostatic stress responses, thereby promoting longevity. To test this, we first examined ER UPR activity and found that *pcyt-1* RNAi was sufficient to activate PEK-1 in WT animals (Fig. 6*C*). In addition, as PC reduction *via sams-1* deficiency has been reported to activate the IRE-1/XBP-1 reporter *hsp-4p*::GFP (34), we confirmed that *pcyt-1* RNAi also induced *hsp-4p*::GFP (Fig. 6*D*). Thus, *pcyt-1* RNAi activates both IRE-1/XBP-1 and PEK-1 pathways. In line with this, *pcyt-1* RNAi enhanced resilience to the ER stressor dithiothreitol in WT animals (Fig. 6*E*).

Given that *pcyt-1* RNAi sufficiently improves cytosolic proteostasis (Fig. 3), we also tested animal resilience to heat shock, a typical proteostatic stress, and indeed observed that *pcyt-1* RNAi increased thermotolerance (Fig. 6*F*). Lastly, as both ER and heat stress resistance are closely linked to aging, we examined animal lifespan and found that *pcyt-1* RNAi significantly extended lifespan (Fig. 6*G*). Together, these findings suggest that PC reduction alone is sufficient to exert multiple health-improving effects and ultimately promotes longevity.

## Discussion

**Figure 7:**
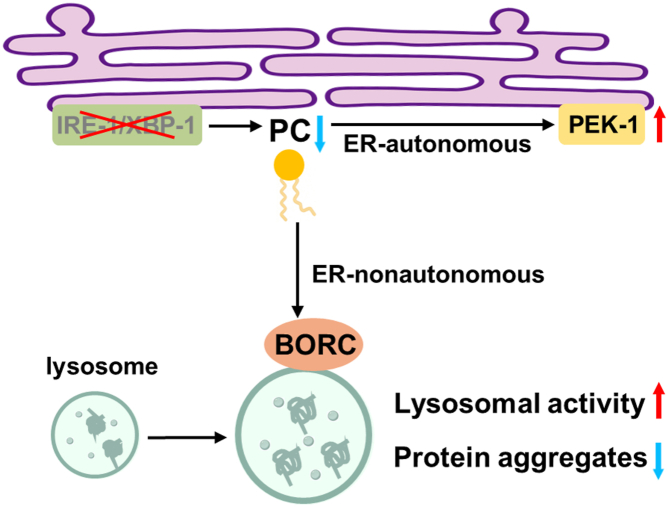
**Proposed working model.** In animals deficient in IRE-1/XBP-1 branch of the ER UPR, PC levels are reduced. This lipid change triggers lysosomal enlargement *via* the BORC subunit SAM-4, which is associated with an enhanced lysosomal function. Consequently, cytosolic proteostasis is improved. In parallel, PC reduction activates the PEK-1 pathway in an ER-autonomous manner. Together, these adaptations contribute to the maintenance of cellular proteostasis under IRE-1/XBP-1 deficiency.

While numerous studies have elucidated how cells respond to ER stress *via* the UPR machinery, the question of whether defects in the UPR itself also constitute a stress that can be sensed by cells and trigger an adaptive response remains unclear. In this study, we reveal such a response, suggesting that the proteostasis surveillance machinery itself, including the UPR, is also under surveillance (Fig. 7). Given the central role of the ER UPR in maintaining cellular proteostasis, this mechanism ensures that cells do not collapse due to a single UPR dysfunction.

More importantly, this response is not solely an ER-autonomous mechanism, but involves communication with cytoplasmic lysosomes. Consistent with our findings, extensive research has explored how declines in mitochondrial proteostasis activate cytoplasmic protein quality control systems (44, 45, 46, 47). These studies together suggest that cellular proteostasis is a holistic system, where protein quality control systems across individual subcellular compartments communicate with one another. Dysfunction in one system can affect the entire cell, while improvements in one system can benefit the whole cell. Our understanding of the interactions between subcellular proteostatic systems remains in its early stages, and further research is needed to fully elucidate this complex network of communication.

Notably, we did not observe an increase in polyQ aggregation in WT animals treated with *hsf-1* RNAi, which appears inconsistent with previous studies. We speculate that this discrepancy might arise from the different time points at which polyQ aggregation was assessed. Earlier reports have shown that *hsf-1* RNAi increased polyQ aggregation in WT animals at either Day 3 or Day 5 of adulthood (48, 49, 50). In contrast, we observed that *hsf-1* RNAi did not affect polyQ aggregation in WT animals at day 8 of adulthood. This suggests that the loss of *hsf-1* may accelerate polyQ aggregation during early adulthood, but by day 8, both WT and *hsf-1* RNAi-treated worms eventually reach similar levels of polyQ accumulation.

We observed lysosomal enlargement in *xbp-1* mutants, leading to the identification of BORC, a key regulator of lysosomal size, as the mediator of *xbp-1*-dependent control over lysosomal morphology and function. While the precise mechanism by which BORC regulates lysosomal function remains unclear, we propose that its functional effects may operate through morphological remodeling, such that BORC-induced lysosomal enlargement potentiates degradative capacity. This potentiation may occur through increased luminal hydrolase content within individual enlarged lysosomes, consequently enhancing cargo degradation efficiency upon autophagosome-lysosome fusion. This hypothesized morphology-function coupling requires further systematic validation.

PC mediates both ER-autonomous and non-autonomous responses to UPR defects, ensuring that these responses are efficiently coordinated. More importantly, this provides a mechanism by which both the ER UPR and lysosomes can be activated simultaneously. Consistent with this, we demonstrate that reducing PC levels achieves this coordination, enhancing the resistance of nematodes to proteostatic stresses and extending lifespan. The metabolism or content of PC in mammals can be modulated through genetic, pharmacological, or dietary interventions. Whether lowering PC could have a similar health-promoting effect in mammals remains an important question worth exploring. However, given the essential role of phosphatidylcholine in normal cellular function, any efforts to reduce its levels should be approached with caution to avoid unintended consequences.

## Experimental procedures

### *C. elegans* strains and maintenance

*C. elegans* were cultured on standard nematode medium (NGM) seeded with *E. coli* OP50 to 1 (51). The following strains were provided by *Caenorhabditis* Genome Center: wild-type N2 Bristol, VC2428[*sams-1(ok2946)*], AM140[*unc-54p*::Q35::YFP], SJ4005[*hsp-4p::gfp*], DA2123[*lgg-1p::gfp::lgg-1*], MAH215[*lgg-1p::mCherry::gfp::lgg-1*], SJ17[*xbp-1(zc12)*], RB545[*pek-1(ok275)*], and RB772[*atf-6(ok551)*]. XW5399[*ced-1p::nuc-1::mCherry*] and XW19180[P_*hs*_NUC-1::pHTomato] were provided by Dr Xiaochen Wang. Strains expressing SAM-4::GFP and polyQ35::GFP in the hypodermis were generated in the author’s laboratory by cloning the *dpy-7* promoter region along with the full-length *sam-4* and polyQ35 sequences into pPD95.79 vector. Double mutants were generated by standard genetic techniques.

### RNA interference treatment

For the RNAi experiment, HT115 bacteria containing specific dsRNA-expression plasmids (Ahringer library) (52) were cultured overnight at 37 °C in LB medium supplemented with 100 μg/ml carbenicillin and seeded onto NGM plates containing 5 mM IPTG.

### qRT-PCR

qRT-PCR was performed as previously described (53). Briefly, day 1 adult worms were collected, washed in M9 buffer, and homogenized in Trizol reagent (Life Technologies). RNA was extracted according to the manufacturer's protocol, and DNA contamination was removed using DNase I (Thermo Fisher Scientific). The RNA was then reverse transcribed to cDNA using the RevertAid First Strand cDNA Synthesis Kit (Thermo Fisher Scientific). Quantitative PCR was carried out using SYBR Green (Bio-Rad), and data were collected with CFX Maestro Software. Primer sequences were listed in Table S2.

### Immunoblotting

Day 1, adult worms were collected and sonicated in RIPA buffer containing 1 mM DTT and proteinase inhibitor (Beyotime) before boiling and loading. Antibodies against GFP (Santa Cruz, SC-9996, 1:2000), mCHEERY (Abcam, ab167453, 1:2000), Phospho-eIF2α (Cell Signaling Technology, #9721, 1:1000), and TUBULIN (Sigma, T9026, 1:4000) were used. The specificity of these antibodies has been validated by the manufacturers. The images were quantified by ImageJ 1.54 m software.

### Cytosolic protein aggregation and associated motility

Protein aggregation in AM140 worms was assessed by measuring the number of polyQ35::YFP foci in each worm. To assess animal motility, a circle with a diameter of 20 mm was drawn at the bottom of NGM plates. Day 8 adult worms were placed at the center of the bacterial food, and after 4 h, the percentage of worms that crawled out of the circle was recorded as a measure of motility.

### Lysosomal assays

To analyze GFP or mCHERRY fluorescence, day 1 adult worms were paralyzed with 1 mM levamisole and mounted on slides for fluorescent microscopy. Lysosomal size, as indicated by NUC-1::mCHERRY or mCHERRY::GFP::LGG-1, was quantified using Leica LAS X software. To calculate the GFP/mCHERRY ratio in the mCHERRY::GFP::LGG-1 strain, images from a 10 × 10 μm area per worm were analyzed. To quantify the association between polyQ35::GFP and NUC-1::mCHERRY, we calculated the ratio of mCHERRY signal overlapping with GFP to the total mCHERRY signal within a 10 × 10 μm area. For Magic Red staining (ImmunoChemistry Technologies), the stock solution was prepared according to the manufacturer’s instructions. 2 μl of stock solution was diluted in 48 μl of M9 buffer and added to the surface of the bacterial lawn. Adult worms were then placed on the Magic Red plates and cultivated for 12 h before imaging. To measure lysosomal acidity, P*hs*NUC-1::pHTomato worms were incubated at 35°C for 1 h, followed by a 24-h recovery period before imaging. Fluorescence intensity was used as a measure of lysosomal acidity.

### Quantification of PC, PE, and TAG

Thin-layer chromatography (TLC) was conducted as previously described (54, 55). Approximately 50,000 days-1 adult worms were harvested and rinsed with M9 to eliminate bacteria, then sonicated in 0.25 ml of PBS. Subsequently, a 5 ml mixture of ice-cold chloroform: methanol (1:1) was added and mixed thoroughly. The solution was incubated overnight at −20 °C with periodic agitation to facilitate lipid extraction. Following incubation, 2.2 ml of Hajra’s solution (0.2 M H_3_PO_4_; 1 M KCl) was introduced. The resultant lower organic phase, enriched with lipids, was isolated by centrifugation at 3000 rpm for 1 min. Lipids were dried under nitrogen and reconstituted in chloroform for TLC separation. Silica gel TLC plates were activated by heating at 110 °C for 75 min. Samples, along with lipid standards, were applied to the TLC plates. Chromatography was performed using a solvent mixture of chloroform:methanol:water:acetic acid (65:43:3:2.5) until the solvent front reached three quarters of the plate's height. Following drying, the plate was developed with a new solvent mixture of hexane:diethyl ether:acetic acid (80:20:2) until the solvent front reached the top. The plates were sprayed with 0.005% primuline and visualized under UV light. Spots corresponding to PC, PE, and TAG were scraped into tubes and resuspended in 2.5% H_2_SO_4_ in methanol. After incubation at 80 °C for 1 h, 1 ml of supernatant was mixed with 1.2 ml of hexane and 1.8 ml of water to extract fatty acid methyl esters (FAMEs) for GC-MS/MS analysis. Analysis was performed using a Shimadzu GC-MS-TQ8040 Gas Chromatograph Mass Spectrometer equipped with an SH-Rxi-5sil MS column, and data were collected using GC-MSsolution software. The lipid levels were normalized to protein contents.

### Survival analysis

Lifespan assays were performed as previously described (55). Synchronized L1 worms were added to NGM plates for lifespan analysis at 20 °C. For heat shock resistance, day 1 adult worms were incubated at 35 °C for survival analysis. For DTT resistance, day 1 adult worms were transferred to NGM plates supplemented with 20 mM DTT and incubated at 20 °C for survival analysis.

### Compound supplementation

Supplements were prepared by diluting in M9 buffer and applied to the surface of the bacterial lawn to achieve the following final concentrations: 15 mM choline (Sigma, V900442), 1 mM chloroquine (Sigma, C6628), and 50 μM bortezomib (Solarbio, B7110). L1 stage animals were subsequently transferred to these plates and incubated for 72 h before further analysis.

### Quantification and statistical analysis

Data are presented as mean ± SD. Statistical analysis was performed using GraphPad Prism software. Survival data were analyzed using a log-rank (Mantel-Cox) test. The adult worm motility was analyzed using a Chi-square and Fisher’s exact test. Other data were analyzed by using an ANOVA or *t* test, as indicated in the figure legends. *p* < 0.05 was considered significant.

## Data availability

All data are contained within the article.

## Supporting information

This article contains supporting information.

## Conflict of interest

The authors declare that they have no conflict of interest with the contents of this article.
