# Supplementary material for: Phosphatidylcholine coordinates ER-autonomous and ER-nonautonomous adaptations to unfolded protein response dysfunction
Source: J Biol Chem. 2025 Dec 7;302(1):111026. doi: 10.1016/j.jbc.2025.111026 (PMC12796731; doi:10.1016/j.jbc.2025.111026)
Supplement: Supporting information [file mmc1.pdf]

Phosphatidylcholine coordinates ER-autonomous and ER-nonautonomous adaptations to unfolded protein response dysfunction

Haixiang Tong[#], Wei Li, Pangui Yuan, Xinyu Wang, Shanshan Pang*, and Haiqing Tang*

School of Life Sciences, Chongqing University, Chongqing, 401331, China

*Correspondence: Shanshan Pang, sspang@cqu.edu.cn; Haiqing Tang, hqtang@cqu.edu.cn

Figure S1-S4
Table S1-S2

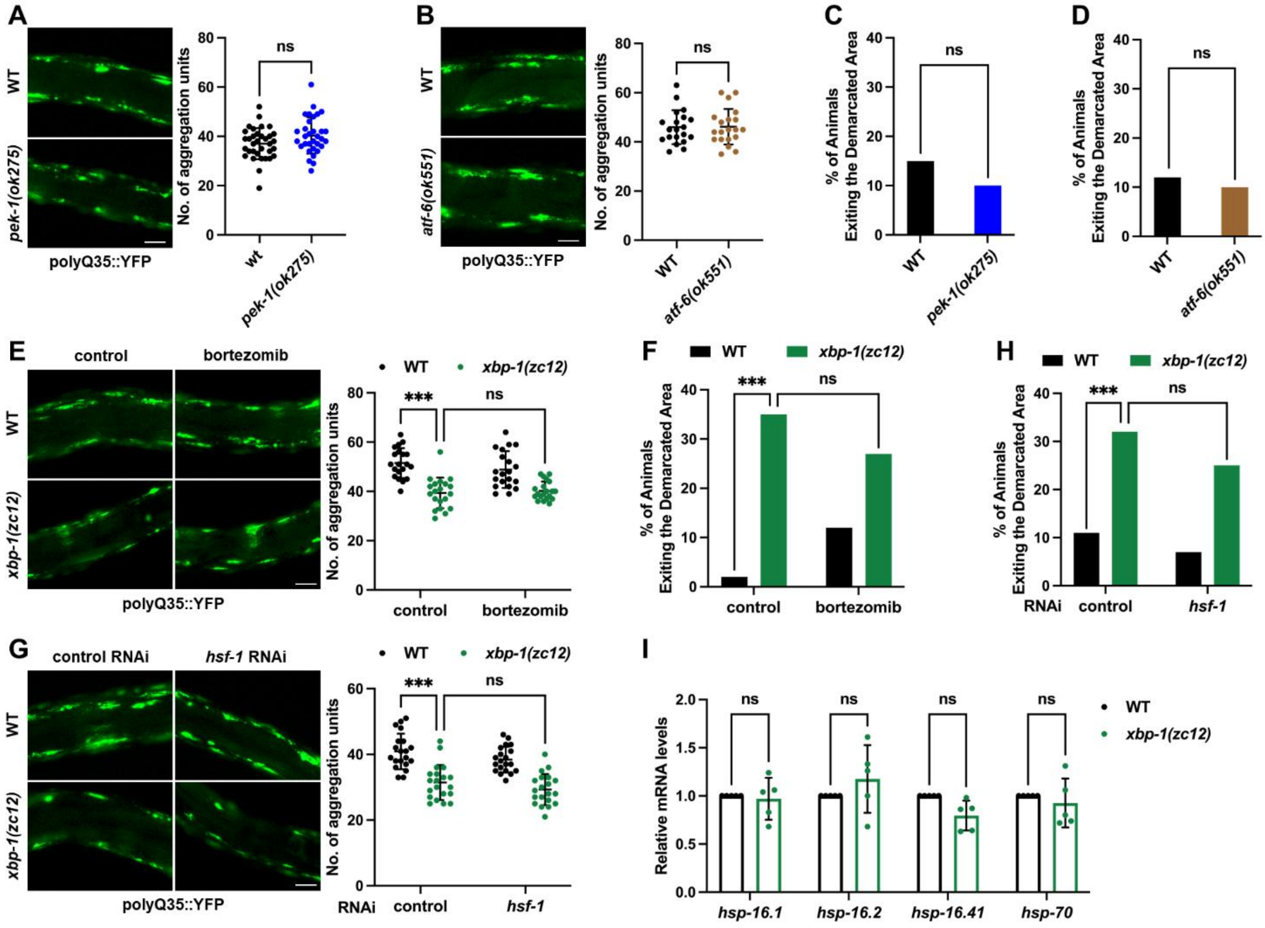

Figure S1. HSF-1 and proteasome are not involved in the regulation of cytosolic proteostasis by *xbp-1* deficiency.

(A-B) Effects of *pek-1(ok275)* mutation (A) and *atf-6(ok551)* mutation (B) on cytosolic polyQ35::YFP aggregation in day 8 adults. n = 25-30 animals.

(C-D) Effects of *pek-1(ok275)* mutation (C) and *atf-6(ok551)* mutation (D) on motility in polyQ35::YFP day 8 adults. n = 80 animals.

(E-F) Effect of bortezomib supplementation on cytosolic YFP aggregation (E, n = 20 animals) and motility (F, n = 80 animals) in day 8 *xbp-1(zc12)* mutants with polyQ35::YFP.

(G-H) Effect of *hsf-1* RNAi on cytosolic YFP aggregation (G, n = 20 animals) and motility (H, n = 80 animals) in day 8 *xbp-1(zc12)* mutants with polyQ35::YFP.

(I) Effect of *xbp-1(zc12)* mutation on the mRNA levels of cytosolic HSP genes. n = 5 independent experiments.

Data are presented as mean ± SD. ***$p < 0.001$. Scale bar = 50 μm. (A, B) were analyzed by unpaired two-tailed *t* test. (C, D, F, H) were analyzed by Chi-square and Fisher's exact test. (E, G)

were analyzed by two-way ANOVA with Tukey's multiple comparisons test. (I) was analyzed by Multiple *t* test with correction for multiple comparisons using the Holm–Sidak method.

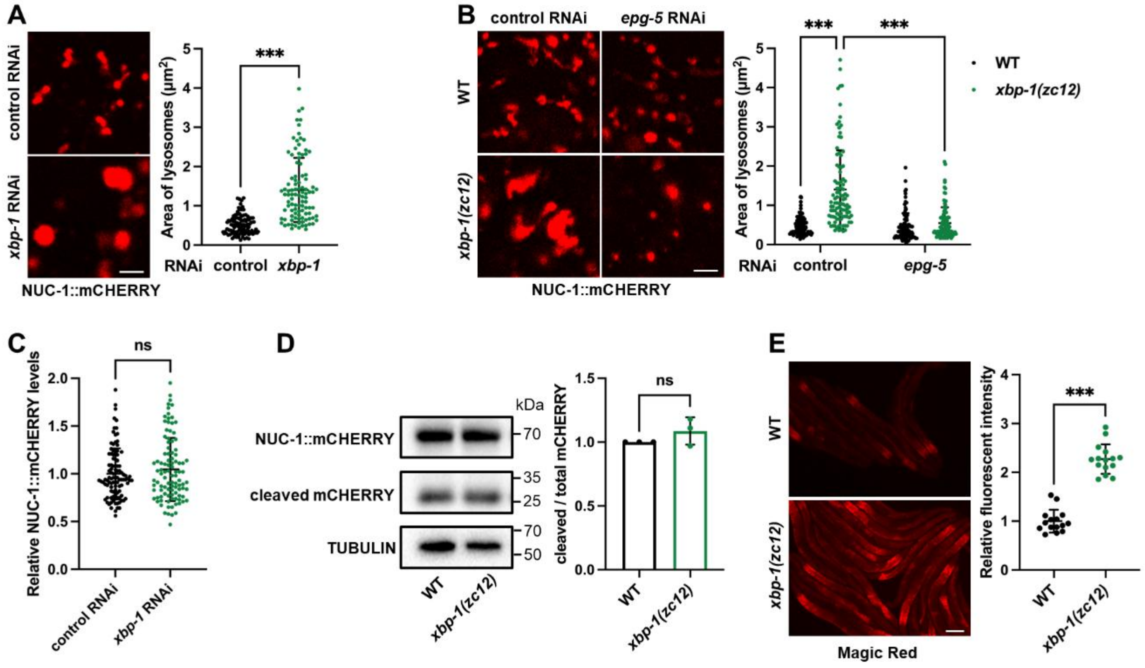

Figure S2. ***xbp-1*** **deficiency affects lysosomal morphology and function.**

(A) Effect of *xbp-1* RNAi on lysosomal morphology and size in day 1 adults. n = 100 lysosomes from 6 worms per group.

(B) Effect of *epg-5* RNAi on lysosomal morphology and size in day 1 *xbp-1(zc12)* mutants. n = 100 lysosomes from 6 worms per group.

(C) Effect of *xbp-1* RNAi on NUC-1::mCHERRY fluorescence intensity in day 1 adults. n = 100 lysosomes from 6 worms per group.

(D) Effect of *xbp-1(zc12)* mutation on NUC-1::mCHERRY cleavage in day 1 adults. n = 3 independent experiments.

(E) Effect of *xbp-1(zc12)* mutation on lysosomal degradation capacity in day 1 adults. n = 15 animals.

Data are presented as mean ± SD. ***$p < 0.001$. Scale bar = 2 μm for panels (A, B); 200 μm for panel (E). (A, C, D, E) were analyzed by unpaired two-tailed *t* test. (B) was analyzed by two-way ANOVA with Tukey's multiple comparisons test.

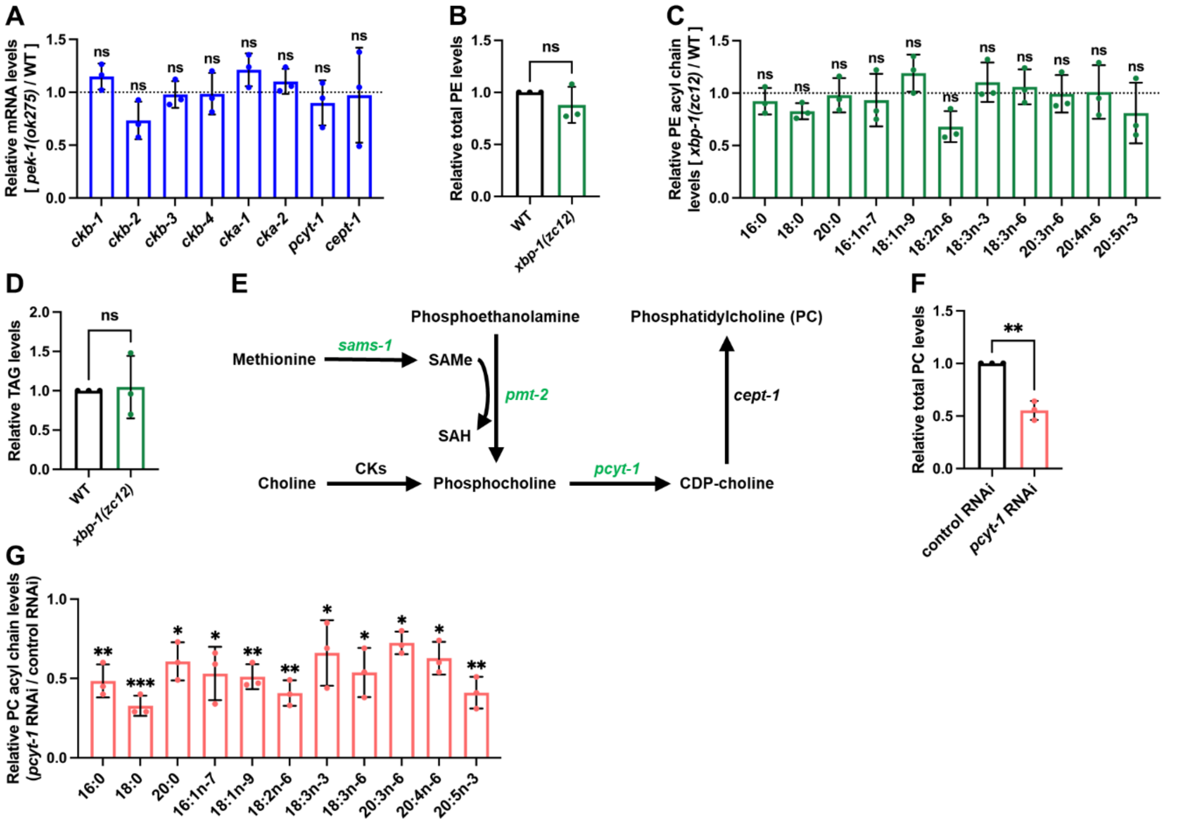

Figure S3. ***xbp-1*** **mutation reduces PC content.**

(A) Effect of *pek-1(ok275)* mutation on the mRNA expression of PC metabolic enzymes in day 1 adults. n = 3 independent experiments.

(B-D) Effect of *xbp-1(zc12)* mutation on the levels of total PE (B), PE acyl chains (C), and TAG (D) in day 1 adults. n = 3 independent experiments.

(E) Schematic of PC metabolism. CK, choline kinase.

(F-G) Effect of *pcyt-1* RNAi on the levels of total PC (F) and PC acyl chains (G) in day 1 adults. n = 3 independent experiments.

Data are presented as mean ± SD. *p < 0.05, **p < 0.01, ***p < 0.001. (A, C, G) were analyzed by Multiple *t* test with correction for multiple comparisons using the Holm–Sidak method. (B, D, F) were analyzed by unpaired two-tailed *t* test.

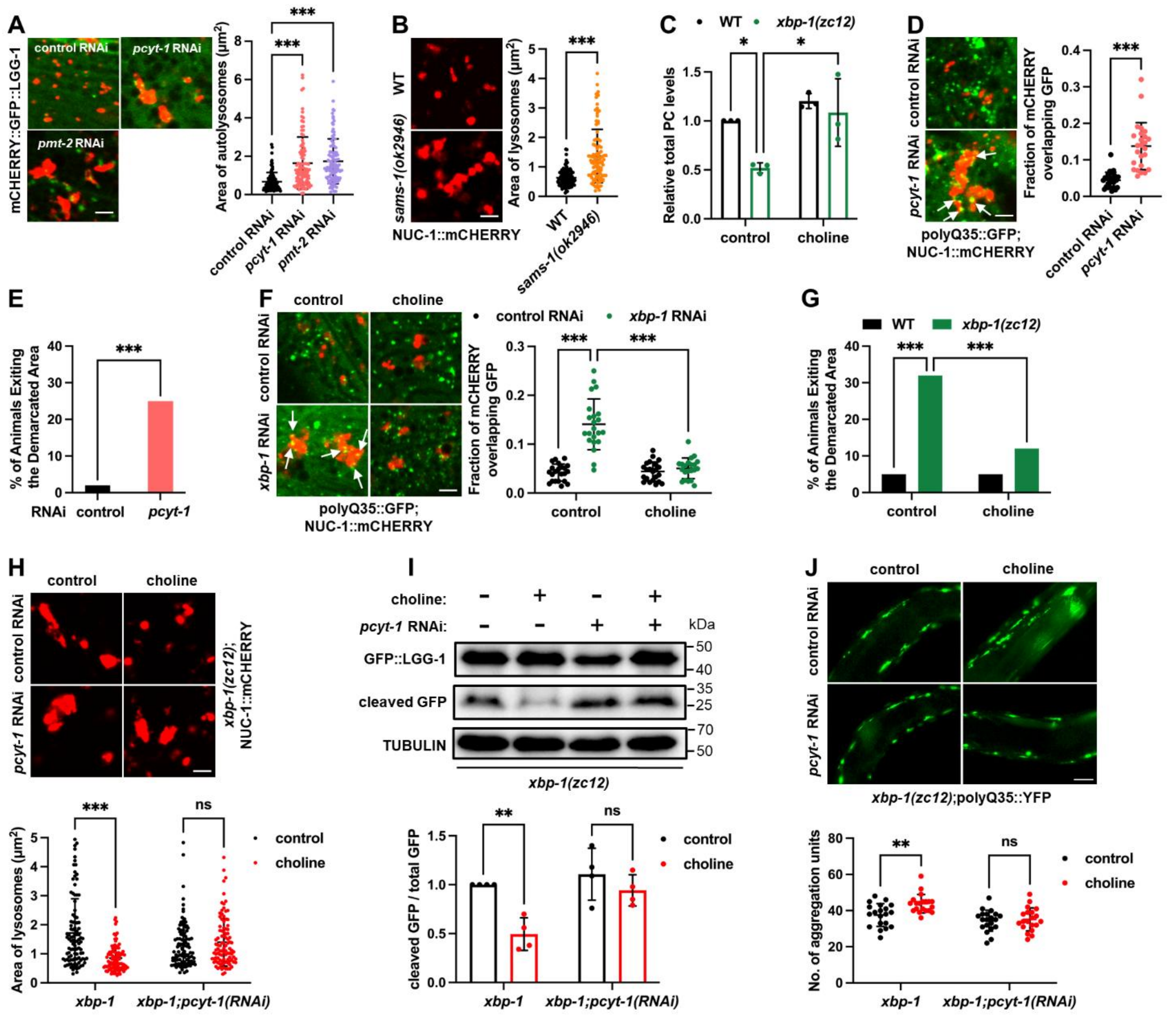

Figure S4. PC reduction causes AL enlargement and improves cytosolic proteostasis.

(A) Effects of *pcyt-1* and *pmt-2* RNAi on the size of ALs in day 1 adults, as indicated by red fluorescence of mCHERRY::GFP::LGG-1. n = 100 ALs from 6 worms per group.

(B) Effect of *sams-1(ok2946)* mutation on lysosomal morphology and size in day 1 adults. n = 100 lysosomes from 6 worms per group.

(C) Effect of choline supplementation on PC levels in day 1 *xbp-1(zc12)* mutants. n = 3 independent experiments.

(D) Effect of *pcyt-1* RNAi on colocalization between polyQ35::GFP and NUC-1::mCHERRY in day 1 adults. n = 25 animals.

(E) Effect of *pcyt-1* RNAi on motility in polyQ35::YFP day 8 adults. n = 80 animals.

(F) Effect of choline supplementation on colocalization between polyQ35::GFP and NUC-

1::mCHERRY in day 1 *xbp-1* RNAi animals. n = 22 animals.

(G) Effect of choline supplementation on motility in polyQ35::YFP day 8 adults with the *xbp-1(zc12)* mutation. n = 80 animals.

(H-I) Effect of choline supplementation on lysosomal size (H) and GFP::LGG-1 cleavage (I) in day 1 *xbp-1;pcyt-1(RNAi)* adults. n = 100 lysosomes from 6 worms per group for (H). n = 4 independent experiments for (I).

(J) Effect of choline supplementation on cytosolic polyQ35::YFP aggregation in day 8 *xbp-1;pcyt-1(RNAi)* adults. n = 20 animals.

Data are presented as mean ± SD. *p < 0.05, **p < 0.01, ***p < 0.001. Scale bar = 2 µm for panels (A, B, D, F, H); Scale bar = 50 µm for panels (J). (A) was analyzed by one-way ANOVA with Dunnett's multiple comparisons test. (B, D) were analyzed by unpaired two-tailed *t* test. (C, F, H, I, J) were analyzed by two-way ANOVA with Tukey's multiple comparisons test. (E, G) were analyzed by Chi-square and Fisher's exact test.

Table S1. Survival data. Repeats 1 were graphed in figures.

Figures	Strain/Treatment	Mean survival time ± SEM (hours/days)	# Worms Censored/Total	Bonferroni P value
6E				
Repeat 1	control RNAi	8.09 ± 0.25	0/67	
	pcyt-1 RNAi	11.18 ± 0.27	0/55	<0.001[a]
Repeat 2	control RNAi	9.11 ± 0.11	0/103	
	pcyt-1 RNAi	10.32 ± 0.24	0/75	<0.001[a]
Repeat 3	control RNAi	9.24 ± 0.18	0/76	
	pcyt-1 RNAi	10.57 ± 0.17	0/70	<0.001[a]
6F				
Repeat 1	control RNAi	9.34 ± 0.1	0/106	
	pcyt-1 RNAi	10.48 ± 0.12	0/109	<0.001[a]
Repeat 2	control RNAi	10.7 ± 0.19	0/70	
	pcyt-1 RNAi	11.99 ± 0.18	0/74	<0.001[a]
Repeat 3	control RNAi	8.72 ± 0.22	0/68	
	pcyt-1 RNAi	10.27 ± 0.21	0/51	<0.001[a]
6G				
Repeat 1	control RNAi	16.14 ± 0.34	0/107	
	pcyt-1 RNAi	18 ± 0.37	0/104	<0.001[a]
Repeat 2	control RNAi	14.49 ± 0.31	2/112	
	pcyt-1 RNAi	16.72 ± 0.37	1/122	<0.001[a]
Repeat 3	control RNAi	16.84 ± 0.35	0/107	
	pcyt-1 RNAi	18.16 ± 0.36	0/102	0.0043[a]

a vs control RNAi

Table S2. qPCR primer sequences.

Primers	Sequences
snb-1 Forward	GCAAGTATTGGTGGAAGA
snb-1 Reverse	ACGATGATGATAATAAGAATGAC
ckb-1 Forward	ATACCACGCTATTGATGT
ckb-1 Reverse	CCGAATATGTGACAGTTG
ckb-2 Forward	AGAATGGAAGAATGTGGATAA
ckb-2 Reverse	AGTGGCTGATGTAGATGA
ckb-3 Forward	ATGTGAATAGGAGTGAAGTG
ckb-3 Reverse	GAAGTCGCAGATGTTGAT
ckb-4 Forward	ACTGGTCAATATGTTCTG
ckb-4 Reverse	TGAGATGAATGAATTAGCA
cka-1 Forward	ATCCGATTATCCGTCAAC
cka-1 Reverse	TTCCATCATCAGCAAGTT
cka-2 Forward	CCTCGTCTTGTGCTCATT
cka-2 Reverse	TCGTAGTCAATAGTCCATTCG
pcyt-1 Forward	AATAATGTCAATGTCTCGTTCA
pcyt-1 Reverse	GTGCCTCCTCTTCTTCTT
cept-1 Forward	GGACGAGTTGTGTATGAA
cept-1 Reverse	GACAGTGATAAGGTTAATGAC
hsp-16.1 Forward	GCAGAGGCTCTCCATCTGAA
hsp-16.1 Reverse	GCTTGAACTGCGAGACATTG
hsp-16.2 Forward	ACCTGAAGATGTAGATGTTG
hsp-16.2 Reverse	TTGCCTGTTGAATTGGAA
hsp-16.41 Forward	ATCGGAACATGGATACTTGA
hsp-16.41 Reverse	AATGGCAGATTTGACAGAAG
hsp-70 Forward	CCGTTGTTGAGGTTGAAG
hsp-70 Reverse	CACAGTAATGACAGCATCC
